# Anti-Inflammatory Principles from *Tamarix aphylla* L.: A Bioassay-Guided Fractionation Study

**DOI:** 10.3390/molecules25132994

**Published:** 2020-06-30

**Authors:** Adel S. Gadallah, Mujeeb-ur-Rehman, Atta-ur-Rahman, Sammer Yousuf, Atia-tul-Wahab, Almas Jabeen, Mahmoud M. Swilam, Shaden A. M. Khalifa, Hesham R. El-Seedi, M. Iqbal Choudhary

**Affiliations:** 1 H.E.J. Research Institute of Chemistry, International Center for Chemical and Biological Sciences, University of Karachi, Karachi-75270, Pakistan; adel.saied22@gmail.com (A.S.G.); mbtk.chem@gmail.com (M.-u.-R.); or ibne_sina@hotmail.com (A.-u.-R.); dr.sammer.yousuf@gmail.com (S.Y.); 2 Department of Chemistry, Faculty of Science, Menoufia University, Shebin El-Kom 32511, Egypt; swilam2040@gmail.com; 3 Dr. Panjwani Center for Molecular Medicine and Drug Research, International Center for Chemical and Biological Sciences, University of Karachi, Karachi-75270, Pakistan; tulwahab@yahoo.com (A.-t.-W.); almas79_jabeen@yahoo.com (A.J.); 4 Department of Molecular Biosciences, The Wenner-Gren Institute, Stockholm University, S-106 91 Stockholm, Sweden; shaden.khalifa@su.se; 5 International Research Center for Food Nutrition and Safety, Jiangsu University, Zhenjiang 212013, China; 6 Department of Biochemistry, Faculty of Sciences, King Abdulaziz University, Jeddah-21589, Saudi Arabia

**Keywords:** *Tamarix aphylla* L., immunomodulatory, reactive oxygen species (ROS), nitric oxide (NO), tumor necrosis factor (TNF-*α*), T-cell proliferation

## Abstract

Natural products have served as primary remedies since ancient times due to their cultural acceptance and outstanding biodiversity. To investigate whether *Tamarix aphylla* L. modulates an inflammatory process, we carried out bioassay-guided isolation where the extracts and isolated compounds were tested for their modulatory effects on several inflammatory indicators, such as nitric oxide (NO), reactive oxygen species (ROS), proinflammatory cytokine; tumour necrosis factor (TNF-*α*), as well as the proliferation of the lymphocyte T-cells. The aqueous ethanolic extract of the plant inhibited the intracellular ROS production, NO generation, and T-cell proliferation. The aqueous ethanolic crude extract was partitioned by liquid-liquid fractionation using *n*-hexane (*n*-C_6_H_6_), dichloromethane (DCM), ethyl acetate (EtOAc), *n*-butanol (*n*-BuOH), and water (H_2_O). The DCM and *n*-BuOH extracts showed the highest activity against most inflammatory indicators and were further purified to obtain compounds **1**–**4**. The structures of 3,5-dihydroxy-4’,7-dimethoxyflavone (**1**) and 3,5-dihydroxy-4-methoxybenzoic acid methyl ester (**2**) from the DCM extracts; and kaempferol (**3**), and 3-hydroxy-4-methoxy-(*E*)-cinnamic acid (**4**) from the *n*-BuOH extract were elucidated by different spectroscopic tools, including MS, NMR, UV, and IR. Compound **2** inhibited the production of ROS and TNF-*α*, whereas compound **3** showed inhibitory activity against all the tested mediators. A better understanding of the potential aspect of *Tamarix aphylla* L. derivatives as anti-inflammatory agents could open the door for the development of advanced anti-inflammatory entities.

## 1. Introduction

Inflammation is tissues’ response to insults such as infections, injuries, burns, and other harmful stimuli, manifested with fever, anorexia, and oedema. It is one of the first innate defence lines and works to remove harmful stimuli and initiate healing processes. Under normal physiological conditions this defence mechanism is vital for health. It could be categorized into acute and chronic. Acute inflammatory processes typically resolve after removal of harmful stimuli; however uncontrolled acute inflammation could become chronic. The chronic inflammatory reaction could lead to devastating ailments, including cardiovascular and neurodegenerative disorders, as well as cancers [1]. The cardinal signs of inflammation are redness, swelling, heat, pain, and loss of tissue function, which are a result of changes in vascular permeability, recruitment of leukocytes, and release of inflammatory mediators. Inflammation activates the cellular signalling pathways regardless of stimuli, sharing common events including recognition of inflammatory stimuli by pattern recognition receptors, activation of signalling pathways, release of inflammatory mediators, and recruitment of leuckocytes to the site of inflammation. Lymphocytes, neutrophils, and macrophages are inflammatory cells that contribute to the process by chemically releasing mediators including acute-phase protein, vasoactive peptides, and cytokines, as well as physically healing tissue damage and restoring function. These chemically released mediators, in addition to other mediators (prostaglandins, histamine, leukotrienes, serotonin, and oxygen- and nitrogen-derived free radicals), interact with the microvasculature and cause interstitial oedema by the increased permeability and cell membrane destruction [2]. The secretion of histamine and interleukins attracts leukocytes and triggers the migration of neutrophils and lymphocytes in a cellular event that recruits the adhesion and firming of the lesion. A cascade of chronic reactions trigger the release of reactive oxygen species (ROS) and nitrogen species [3] in a process later promoted by lymphocyte and monocyte infiltration, fibroblast proliferation, connective tissue formation, and collagen fibre accumulation [4]. Chronic inflammation is critical in initiating devastating disorders, such as Alzheimer’s disease, cardiovascular disease, allergies, autoimmune diseases, metabolic disorders, and cancers [5]. 

The steroidal and non-steroidal anti-inflammatory drugs are the leading conventional therapeutics to relieve inflammation. Despite the successes in alleviating pain and improving fever in most reported cases, they could also cause osteoporosis, peptic ulcers, bronchial spasms, glaucoma, cataract, adrenal failure, and a general decrease in immunity. The side effects of these drugs hamper their long-term utilization and warranted the clinical use of less toxic solutions (e.g., natural products) with reduce adverse reactions [6]. Thus, there is a call for a safe, efficient, and economically affordable alternative therapeutics. 

Natural products have a fundamental role in drug discovery and research [7]. Natural products, including primary or secondary metabolites from plants and animals, marine organisms and microorganisms, contribute to the high diversity of biologically generated compounds and represent potential scaffolds for drug design and development. Approximately 28% of all approved medicines and 80% of anticancer drugs are either natural compounds or are based on natural product in one form or another [8]. Plant-derived drugs are utilized to treat several diseases, such as anti-inflammatory conditions, cancer, and osteoporosis [9,10,11,12]. *Tamarix aphylla* L., a medium sized tree widely distributed in Africa; the Middle East; and parts of Southern and Western Asia [13], belonging to the family Tamaricaceae. *T. aphylla* L., has several synonyms, such as *Tamarix orientalis* Forssk, *Thuja aphylla* L., and *T. articulate* Vahl. It is also known as; Quranic name: Athel, Arabic name: Abal, Tarfaa, Ghaz, Athel. English name: Athel tamarisk [14]. *Tamarix* species possess various classes of compounds, i.e., tannins, phenolic acids, flavonoids, and sulfur-containing compounds, and are traditionally used to cure skin-, spleen-, and eye-related conditions, toothaches, enteritis, stomach aches, animal bites, and poisoning [12,15]. *Tamarix aphylla* L., is a promising natural source known for its metabolite diversity and high potential for medicinal and pharmaceutical leads. The aqueous ethanolic extract of *T. aphylla* L. galls has been recommended as an anti-inflammatory and antipyretic mediator [16], therefore, in the present study, we tested this notion by investigating the constituents of the aerial parts of *T. aphylla* L. and evaluated their anti-inflammatory in vitro activity.

## 2. Results

The anti-inflammatory activities of the crude aqueous ethanol extract and the sub-extracts from liquid-liquid fractionation (*n*-C_6_H_6_, DCM, EtOAc, *n*-BuOH, and H_2_O) of *T. aphylla* L. were investigated on various innate and adaptive immune parameters including the production of intracellular reactive oxygen species (ROS), nitric oxide (NO), the proinflammatory cytokine TNF-α, and the proliferation of T-cells. The different cellular models were used to determine the effect of inflammatory responses in both innate and adaptive immune cells. The peripheral blood lymphocytes containing cells of both innate and adaptive immunity (myeloids and lymphoids) were used for an initial screen. The monocytes (THP-1) and macrophages (J774.2) (monoblast lineage) were used as representative cells for innate immunity because these cells of myeloid origin secrete inflammatory cytokines. T-lymphocytes of lymphoid origin were used as representative cells for adaptive immunity.

### 2.1. Reactive Oxygen Species (ROS)

In the present study, the effect of *T. aphylla* L. constituents against myeloperoxidase-dependent reactive oxygen species (ROS) produced by human whole blood phagocytes was examined. ROS are induced as proinflammatory molecules upon pathogen recognition. Macrophages along other cells react by releasing ROS, nitric oxide synthase (NOS), cyclooxygenase (COX)-2, and tumour necrosis factor-alpha (TNF-α) to combat the inflammatory insults. Our results showed that the EtOH: H_2_O, DCM, EtOAc, and *n*-BuOH extracts inhibited the intracellular ROS generated by zymosan-activated whole blood phagocytes. The EtOAc extract was the most potent inhibitor, showing a dose-dependent inhibition where 100% inhibition was recorded at 250 µg/mL, and the IC_50_ value was found to be 12.0 ± 0.6 µg/mL as compared to ibuprofen as a standard drug (IC_50_ = 11.2 ± 1.9 µg/mL) (see Table 1). The EtOH: H_2_O, DCM, EtOAc, and *n*-BuOH extracts inhibited the ROS with IC_50_ values of 16.7 ± 0.6, 19.2 ± 1.8, 12.0 ± 0.6, and 19.8 ± 3.5 µg/mL, respectively. Compounds **2** and **3** potently inhibited the ROS with 94.8% and 98.8% inhibition at a concentration of 100 µg/mL, whereas compound **1** exhibited a low level of inhibition. Compound **4** was found to be inactive (see Table 1). 

### 2.2. Nitric Oxide (NO)

The EtOH:H_2_O, DCM, and *n*-BuOH extracts inhibited the generation of nitric oxide (NO) in lipopolysaccharide-activated J774.2 cells. The DCM extract exhibited the most potent inhibition with IC_50_ = 14.0 ± 0.6 µg/mL (for comparison, the standard, L-NMMA (NG monomethyl L-arginine acetate) showed IC_50_ = 24.2 ± 0.8 µg/mL). Compound **3** inhibited the NO and showed dose-dependent inhibition whereas 100% inhibition was obtained at 50 µg/mL, and the IC_50_ value was found to be 2.4 ± 0.3 µg/mL. The EtOAc extract showed no inhibitory activity. The EtOH: H_2_O, DCM, and *n*-BuOH extracts inhibited the generation of nitric oxide (NO) with IC_50_ = 29.5 ± 1.7, 14.0 ± 0.6, and 27.2 ± 0.07 µg/mL, respectively.

### 2.3. The Proinflammatory Cytokine TNF-α

The DCM and *n*-BuOH extracts inhibited the production of TNF-α by LPS-activated THP-1 cells, and the other two extracts were inactive (see Table 1). The DCM and *n*-BuOH extracts inhibited TNF-α production with IC_50_ = 3.7 ± 0.3 and 44.9 ± 8.9 µg/mL, respectively. Compound **2** inhibited the production of the proinflammatory cytokine TNF-α with 79.87% inhibition at 50 µg/mL and an IC_50_ value of 12.6 ± 0.2 µg/mL. Compound **3** displayed a significant activity of this cytokine with 99.3% inhibition at 50 µg/mL and an IC_50_ value of 5.5 ± 1.1 µg/mL.

### 2.4. The Proliferation of T-Cells

The EtOAc, *n*-BuOH and EtOH: H_2_O, extracts suppressed the phytoheamagglutinin (PHA)-induced proliferation of T-cells to a great extent, whereas the DCM extract showed no inhibition (see Table 1). The EtOH: H_2_O, EtOAc, and *n*-BuOH extracts suppressed the proliferation of T-cells (IC_50_ = 13.8 ± 2.4, 43.3 ± 1.2, and 8.1 ± 0.3 µg/mL, respectively). Compound **3** also inhibited the proliferation of T-cells (IC_50_ = 22.9 ± 1.5 µg/mL).

### 2.5. Bioassay Guided Fractionation

**Figure 1:**
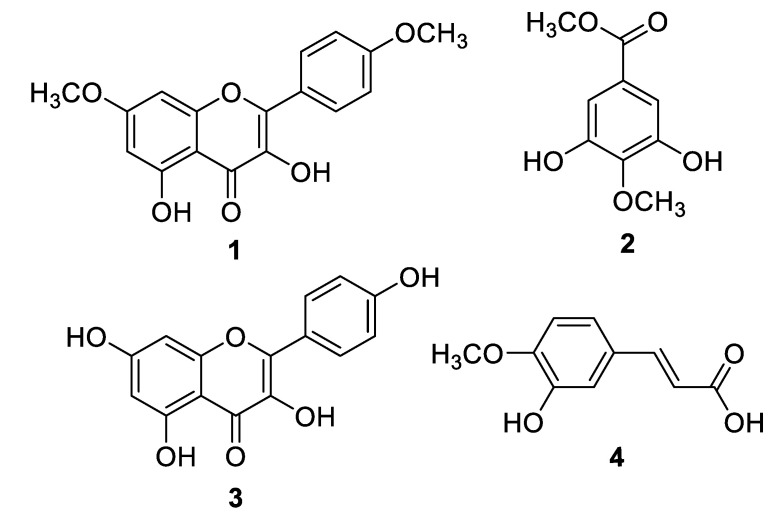
Structures of Compounds **1**–**4**.

Compounds **1** and **2** (Figure 1) were isolated from the DCM extract, and compounds **3** and **4** were isolated from the *n*-butanol extract. By comparing the spectroscopic data of the isolated compounds with the literature data, they were identified as 3,5-dihydroxy-4′,7-dimethoxyflavone (**1**) [17], and 3,5-dihydroxy-4-methoxybenzoic acid methyl ester (**2**) [18]. 

**Figure 2:**
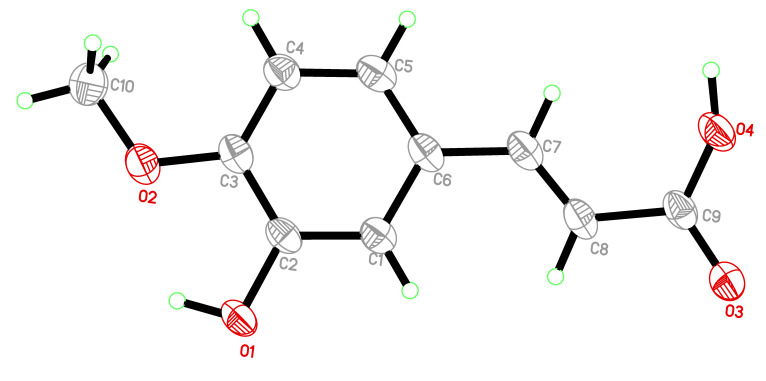
Single-Crystal X-ray ORTEP. diagram of *trans*-isoferulic acid (**4**).

Likewise, compounds **3** and **4**, obtained from the *n*-BuOH extract, were identified as kaempferol (**3**) [17], and *trans*-isoferulic acid ((*E*)-3-hydroxy-4-methoxycinnamic acid) (**4**) [19]. Single-Crystal X-ray diffraction, as shown in Figure 2, was also used to confirm the structure of compound **4**. The isolated compounds **1**, **2**, **3**, and **4** were initially assessed for their inhibitory effect on intracellular ROS. The active compounds **2** (4-methoxy methyl gallate) and **3** (kaempferol) were further evaluated for their inhibitory potential on inflammation. Compounds **2** and **3** showed a potent anti-inflammatory and immunomodulatory effects, whereas compounds **1** and **4** were found to be inactive in the assay system.

The IC_50_ (µg/mL) values were calculated using three doses of each extract and compound. Values are presented as the mean ± SD of triplicates. In the first column, ROS inhibition was described, and ibuprofen was used as standard. For remaining assays in the next three columns of Table 1, NT means not tested, and the symbol (−) was shown when ibuprofen is not a standard drug; however, for NO activity, L-NMMA (NG-monomethyl-L-arginine) is standard (Table 1). 

## 3. Discussion

### 3.1. Tamarix aphylla *L.* Extracts

The extracts and secondary metabolites from the aerial parts of *T. aphylla* L. were evaluated for their phytochemical and immunomodulatory potential. All extracts showed immunosuppressive activities on the tested parameters to various extents. EtOH: H_2_O extracts of *T. aphylla* L. potently inhibited the ROS production and NO generation with an IC_50_ value of 16.7 ± 0.6 and 29.5 ± 1.7 µg/mL, respectively, whereas it demonstrated only moderate activity against TNF-α production and T-cell proliferation. Ali et al., reported in vivo wound healing activity of 70% ethanolic extracts of *T. aphylla* L. leaves with significant antioxidant activity and mild suppression of TNF-α production [10]. 

In our study, The DCM and *n*-BuOH extracts of *T. aphylla* inhibited ROS, NO, and TNF-α production, whereas the *n*-BuOH extract inhibited the proliferation of T-cells. Taken together, the critical role of inflammation in eliminating pathogens is often overshadowed by the continuous inflammatory processes that eventually causes more damage which can occasionally lead to cancer. Thus, the early control of inflammation with the aid of anti-inflammatory agents could shorten the course and limit long-term consequences. Natural products, such as *Tamarix aphylla* L. seem to trigger innate immunity and apoptosis/programmed cell death. The innate and adaptive immunities are stimulated by loads of signals, both vascular and cellular, before internalization and pathogen ingestion. Signalling proteins and molecules are generated *via* immune system activation. T-lymphocytes interact with other immune cells (monocytes, neutrophils, dendritic cells, and natural killer cells) and other mediators, including reactive oxygen species, nitric oxide, macrophage inflammatory protein-2, and transforming growth factors. This process initiates a cascade of pathways that induce interleukins and TNF-α before successful healing and restoration [20,21]. Under this condition, acute/short-term (chemokines) and chronic/long-term (lymphocytes) processes and attenuation of the continuous systemic immune response are limited. 

### 3.2. Purified Compounds

The basic skeleton of flavonoids and their flavones subgroup consist of three rings with the characteristic unsaturated C ring, C-4 carbonyl group, and hydroxyl group. Hydroxyl and aromatic rings have different numbers and positions in the flavonoid molecules thus exhibiting a variant degree of activity [22] that explains its antioxidant and anti-inflammatory effects. The current paper discusses the isolation and structure elucidation of four bio-active constituents. Two of them are flavones as characterized by NMR, UV, and Mass Spectroscopy. The spectral data matched the reported ones in the literature screening. The other two were methyl ester and cinnamic acid derivatives, which were also identified in the same way.

#### 3.2.1. Kaempferol-7,4’-dimethyl ether (**1**)

Compound **1** was reported from several medicinal plants, including *Renealmia thyrsoidea*, *Hedychium thyrsiforme* Sm., *Boesenbergia kingie,* and *Boesenbergia longiflora* [23,24,25,26]. 3,5-Dihydroxy-4’,7-dimethoxyflavone has demonstrated significant inhibition to ROS generation. Sudsai et al., reported its antioxidant activity *via* anti-DPPH (2,2-diphenyl-1-picrylhydrazyl) and free radical scavenging activity [26]. The compound was inactive when testing NO and TNF-α production, which is in agreement with previous studies [25,26]. The methylation of flavone methyl groups could augment bioavailability based on gastrointestinal stimulation [27].

#### 3.2.2. Methyl 4-*O*-methylgallate (**2**)

The inhibitory effect of compound **2** on the production of ROS and TNF-*α* is correlated with the previous study by Correa et al., 2016, where methyl gallate was reported to attenuate the inflammatory arthritis and inhibit various inflammatory mediators by interacting with multiple molecular targets, mainly *via* the phosphatidylinositol-3-kinase (PI3K) signalling pathway. Various Toll-like receptor (TLR) stimuli are responsible for the activation of PI3K and the subsequent phosphorylation of protein kinase B (AKT) [28].

#### 3.2.3. Kaempferol (**3**)

Kaempferol (**3**), a widely distributed flavonol in nature, is biologically active, which acts as radical scavenging, anti-inflammatory, and antimicrobial agent. Kaempferol (**3**) affects various inflammation-related diseases, such as cancers, cardiovascular diseases, and neurodegenerative maladies [29]. These activities, characteristic of kaempferol, are due to its ability to inhibit the production of inflammatory mediators, including proinflammatory cytokines and chemokine expression [30]. In addition to their potent in vitro antioxidant activities, the in vivo antioxidant effects of most flavonoids depend on their absorption and bioavailability [31]. Compound **3** (kaempferol) potently inhibited the production of ROS, TNF-*α*, and NO, whereas it moderately inhibited the proliferation of T-cells in the assay system. Various classes of flavonoids are reported for their therapeutic potential. The inhibitory effects of most flavonoids on inflammation is through inhibiting the production of inflammatory cytokines and by blocking either the MAPK (mitogen-activated protein kinase) or NFκB (nuclear factor kappa B) and AP-1 (Activator protein-1) activation pathways [32]. The antioxidant activity of kaempferol (**3**) is superior to that of ibuprofen, as observed in our assay (Table 1). The higher inhibitory activity of kaempferol (**3**) on intracellular ROS may be due to conformational changes provided by the flexibility of its non-planar structure. This flexibility increases its ability to pass through the plasma membrane [33]. Kaempferol (**3**) is reported to exert its anti-inflammatory effect by attenuating the activity of AP-1 by inhibiting c-jun mRNA expression and JNK [34].

#### 3.2.4. *Trans*-Isoferulic Acid (**4**)

Cinnamic acid derivatives have been isolated and identified from several medicinal plants. Cinnamic acids are phenolic compounds naturally occurring in vegetables, flowers, and fruits, and are enriched in daily diets. The local and general uses of cinnamic acid are reported as non-irritating and safe. Cinnamic acid derivatives are suggested to possess an anti-inflammatory effect [35,36,37]. In agreement, we observed that *trans*-isoferulic acid ((*E*)-3-hydroxy-4-methoxycinnamic acid) was a potent inhibitor of TNF-α production. 

## 4. Materials and Methods

### 4.1. General Information

Column chromatography (CC) was performed using silica gel (230–400 mesh, E. Merck, Darmstadt, Germany). Thin-layer chromatography (TLC) separations were carried out on pre-coated silica gel sheets (60 F_254._ E. Merck), and the compounds were detected under UV light at 254 and 366 nm. Then, the plates were sprayed with ceric sulfate in 10% H_2_SO_4_ and were gently heated using a heat gun until the development in colour was observed. Recycling preparative HPLC (RP-HPLC (JAI LC-908W, Japan Analytical Industry Co. Ltd., Tokyo, Japan) with a YMC ODS H-80 or L-80 column (YMC, Tokyo, Japan) was used for the final purification. A UV-3200 spectrophotometer (Tokyo, Japan) was used to obtain the UV spectra, and the IR spectra were obtained from KBr discs on an A-302 spectrometer (JASCO, Tokyo, Japan). The EI-MS spectra were acquired on a EI (LR) JEOL MS ROUTE 600H, (JEOL Ltd, Tokyo, Japan). HREI-MS were recorded on a EI (HR) MAT 95XP ThermoFinnigan, Germany. The NMR spectra were acquired on AV-300, AV-400, and AV-500 instruments (Bruker, Switzerland). All chemical shifts values are displayed in ppm (δ). The ^1^H-NMR, COSY, HSQC, and HMBC spectra were recorded at 400 MHz, while the ^13^C NMR spectra were obtained at 100 MHz. The coupling constants (*J*) are estimated in Hertz.

### 4.2. Plant Material

Aerial parts of *Tamarix aphylla* L. were collected from medium-aged trees from El-Nubaria city, 75 km Cairo-Alexandria desert road, Egypt, in February and March 2016. The plant material was identified by Prof. Dr. Zaki Turki from the Department of Botany, Faculty of Science, Menoufia University, Egypt. A voucher specimen (ETA. 2,3-2016) was deposited in the herbarium of the Department of Botany, Faculty of Science, Menoufia University, Shebin El-Kom, Egypt.

### 4.3. Extraction and Isolation

The dried and ground plant material (*Tamarix aphylla* L., 1.8 kg) was extracted using a Soxhlet apparatus with 80% EtOH: H_2_O at 70–80 °C. The extract was concentrated *in vacuo* at 45 °C and freeze dried for 8 h to afford 300 g of semi-solid dark material (crude extract). The crude extract was suspended in distilled H_2_O and then successively partitioned between *n*-hexane, DCM, EtOAc, *n*-BuOH, and H_2_O to generate the corresponding sub-extracts. The DCM sub-extract (20 g) was loaded onto a normal-phase silica (NPS) gel column (70 × 5.6 cm) and was eluted with gradient mobile phase system of DCM: *n*-Hexane (10, 20, … 90%), DCM, and MeOH: DCM (1, 2, 3, … 15%) to afford 12 major fractions (1–12); fraction 7 (980 mg) was evaluated on a TLC with 60% DCM: *n*-hexane as the eluent system. TLC indicated that this fraction was pure compound **1**.

Fraction 9 (4.3 g) was loaded onto an NPS column (75 × 3.5 cm) and eluted with a gradient mobile phase system of EtOAc: *n*-hexane (2, 4, 6, 8, 10, 12, 14, 16, 18, 20, 25, … 40%) to obtain six sub-fractions, while sub-fraction 5 (400 mg) was loaded onto a Sephadex LH-20 column (100 × 2.5 cm) using DCM: MeOH (2:1) as an eluent system to obtain compound **2** (11 mg). Furthermore, RP-HPLC (6:4 ACN: H_2_O, a flow rate of 4 mL/min) was used for further purification of 90 mg of the impure compound, which led to isolation of compound **2** (60 mg) as a pure secondary metabolite (R_t_ = 20 min).

The BuOH sub-extract (32 g) of *T. aphylla* L. was loaded onto an NPS column (150 × 6.9 cm) and eluted with DCM and 0.5% to 25% MeOH: DCM into 95 flasks (250 mL). Flasks 24–27 were combined and concentrated *in vacuo* at 45 °C to obtain 1.1 g of material. This material was loaded onto a Sephadex LH-20 column (75 × 4 cm) and eluted with 100% MeOH to obtain compounds **3** (62 mg) and **4**. Compound **4** (50 mg) was recrystallized from HPLC grade MeOH.

#### 4.3.1. Kaempferol-7,4’-dimethyl ether (**1**)

Yellow, amorphous powder; UV (MeOH) λ_max_ nm: 211, 233, 269, 327, 368; IR (KBr) ν_max_ 3314, 2922, 2848, 1836, 1743, 1657, 1596, 1507, 1463, 1355, 1318, 1258, 1220, 1162, 1033 cm^−1^; ^1^H-NMR (CDCl_3_, 400 MHz): δ 11.71 (1H, s, H-O-5), 8.14 (2H, d, *J* = 9.0 Hz, H-2′ and H-6′), 7.01 (2H, d, *J* = 9.0 Hz, H-3′ and H-5′), 6.58 (1H, s, H-O-3), 6.46 (1H, d, *J* = 2.1 Hz, H-8), 6.35 (1H, d, *J* = 2.1 Hz, H-6), 3.87 (3H, s, H_3_CO-7), 3.86 (3H, s, H_3_CO-4′). ^13^C-NMR (CD_3_OD, 100 MHz): δ 175.2 (C-4), 165.7 (C-7), 161.1 (C-4′), 160.8 (C-5), 156.8 (C-9), 145.7 (C-2), 135.7 (C-3), 129.4 (C-2′ and C-6′), 123.2 (C-1′), 114.1 (C-3′ and C-5′), 103.9 (C-10), 97.9 (C-6), 92.2 (C-8), 55.8 (CH_3_O-7), 55.4 (CH_3_O-4′) HREI-MS: *m*/z 314.078 calculated for C_17_ H_14_ O_6_ (Calcd. 314.079)

#### 4.3.2. Methyl 4-*O*-methylgallate (**2**)

Very thin colourless needles; UV (MeOH) λ_max_ nm: 228, 261; IR (KBr) ν_max_ 3389, 3002, 2950, 2846, 1711, 1595, 1511, 1443, 1376, 1290, 1257, 1169, 1059, 996, 762 cm^−1^; ^1^H-NMR (CD_3_OD + D_2_O, 400 MHz): δ 7.03 (2H, s, H-2 and H-6), 4.61 (2H, s, H-O-3 and H-O-5), 3.84 (3H, s, H_3_C-O-CO-1), δ 3.84 (3H, s, H_3_C-O-4). ^13^C-NMR (CD_3_OD, 100 MHz): δ 168.5 (1-CO-OMe), 151.7 (C-3 and C-5), 141.2 (C-4), 126.5 (C-1), 110.1 (C-2 and C-6), 60.7 (CH_3_-O-4), 52.5 (CH_3_-O-CO-1). HREI-MS: *m*/z, 198.05229 calculated for C_9_H_10_O_5_ (Calcd. 198.0528).

#### 4.3.3. Kaempferol (**3**)

Yellow, amorphous powder; UV (MeOH) λ_max_ nm: 211, 262, 366; IR (KBr) ν_max_ 3350, 2927, 2853, 1837, 1743, 1659, 1612, 1568, 1506, 1452, 1380, 1309, 1251, 1227, 1175, 1088, 882, 823 cm^−1^; ^1^H-NMR (CD_3_OD, 400 MHz): δ 8.06 (2H, d, *J* = 8.8 Hz, H-2′ and H-6′), 6.88 (2H, d, *J* = 8.8 Hz, H-3′ and H-5′), 6.37 (1H, d, *J* = 2.0 Hz, H-8), 6.16 (1H, d, *J* = 2.0 Hz, H-6). ^13^C-NMR (CD_3_OD, 100 MHz): δ 177.3 (C-4), 165.5 (C-7), 162.5 (C-5), 160.5 (C-4′), 158.2 (C-9), 148.0 (C-2), 137.1 (C-3), 130.7 (C-2′ and C-6′), 123.7 (C-1′), 116.3 (C-3′ and C-5′), 105.5 (C-10), 99.3 (C-6), 94.5 (C-8). HREI-MS: *m*/z, 286.0487 calculated for C_15_H_10_O_6_ (Calcd. 286.0477).

#### 4.3.4. *Trans*-Isoferulic Acid (**4**)

Colourless crystals; UV (MeOH) λ_max_ nm: 221, 232, 292, 322; IR (KBr) ν_max_ 3405, 2936, 2845, 2584, 1676, 1622, 1513, 1447, 1359, 1317, 1269, 1209, 1132, 1020, 945, 857, 815. ^1^H-NMR (DMSO, 400 MHz): δ 12.18 (1H, s, H-O-CO), 9.16 (1H, s, H-O-3′), 7.40 (1H. d, *J* = 16.0 Hz, H-3), 7.08 (1H, d, *J* = 2.0 Hz, H-6′), 7.06 (1H, s, H-2′), 6.90 (1H, d, *J* = 8.0 Hz, H-5′), 6.20 (1H, d, *J* = 16.0 Hz, H-2), 3.79 (3H, s, H_3_C-O-4′). ^13^C- NMR (DMSO, 100 MHz): 167.7 (C-1), 149.8 (C-4′), 146.6 (C-3′), 144.1 (C-3), 127 (C-1′), 120.9 (C-6′), 116.2 (C-2), 114 (C-2′), 111.9 (C-5′), 55.6 (CH_3_-O-4′). HREI-MS: *m*/z, 194.0587 calculated for C_10_H_10_O_4_ (Calcd. 194.0579).

### 4.4. Anti-Inflammatory and Immunomodulatory Activities

All studies on cells from human blood were carried out after approval from an independent ethics committee, ICCBS, UoK, No: ICCBS/IEC-008-BC-2015/Protocol/1.0.

#### 4.4.1. Oxidative Burst Inhibition Assay

A luminol-enhanced chemiluminescence assay was used as defined by Helfand et al. 1982 [38] with some modifications. The assay is used to detect intracellular reactive oxygen species produced by the blood phagocytes. In brief, 25 µL of whole blood diluted with HBSS^++^ (Hanks Balanced salt solution, containing calcium chloride and magnesium chloride) (Sigma, St. Louis, MO, USA) was incubated with 25 µL of three different concentrations (1, 10, and 100 µg/mL) of the test compounds, and each concentration was tested in triplicate. Control wells received HBSS^++^ and cells but not the test compounds. Tests were performed in 96-well plates (Costar, Manhattan, NY, USA), and samples were incubated at 37 °C for 15 min in a temperature-controlled luminometer chamber (Labsystems, Helsinki, Finland). After incubation, 25 µL of serum opsonized zymosan (SOZ) (Fluka, Buchs, Switzerland) and 25 µL of the intracellular reactive oxygen species detecting probe luminol, (Research Organics, Cleveland, OH, USA) were added to each well, except the blank wells, which contained only HBSS^++^. The level of ROS in each well was recorded using a luminometer in relative light units (RLU). Ibuprofen was used as the standard drug (IC_50_ = 11.2 ± 1.9 µg/mL) in this assay [30].

#### 4.4.2. Nitric Oxide Inhibition Assay

The assay was performed using mouse macrophages where NO is produced by the cells when activating them *via* bacterial lipopolysaccharides. The mouse macrophage cell line J774.2 (European Collection of Cell Cultures, Salisbury, UK) was cultured in 75-cc flasks (IWAKI, Asahi Techno Glass, Shizuoka, Japan) in DMEM (Sigma-Aldrich, Steinheim, Germany) supplemented with 10% fetal bovine serum (GIBCO, New York, NY, USA) and 1% streptomycin/penicillin. Flasks were kept at 37 °C under humidified air containing 5% CO_2_. Cells were seeded in 96-well plates (10^6^ cells/mL), and nitrite production was induced by adding 30 µg/mL *E. coli* lipopolysaccharide (LPS) (DIFCO Laboratories, Detroit, MI, USA]. The test compounds/extracts were tested at three different concentrations (2, 10, and 50 µg/mL); the plates were incubated at 37 °C in 5% CO_2_ for 48 hrs. Nitrite accumulation in the culture supernatant was measured using the Griess reagent [39]. N^G^ monomethyl L-arginine acetate was used as a standard NO inhibitor (IC_50_ = 24.2 ± 0.8 µg/mL). The results are presented in terms of IC_50_ values that were calculated using a Microsoft Excel-based formula. The compounds showed dose-dependent inhibition, and the highest inhibition was observed at a concentration of 50 µg/mL. 

#### 4.4.3. Proinflammatory Cytokine TNF-α Production and Quantification

Human monocytic leukaemia cells (THP-1) are known for the production of proinflammatory cytokines. The monocytes, after differentiation into macrophages by PMA treatment and stimulation with bacterial lipopolysaccharide, generate TNF-α and IL-1β, among other cytokines. Human monocytic leukemia cells were purchased from the European Collection of Cell Cultures. The cells were maintained in RPMI-1640 containing 5.5 mmol/L glucose (BioM Laboratories, Chemical Division, Kuala Lumpur, Malaysia], 50 µmol/L mercaptoethanol (Merck, Damstadt, Germany), 10% FBS (foetal bovine serum), and 2 mmol/L L-glutamine (PAA Laboratories, GmbH, Pasching, Austria). Cells were grown in 75-cc flasks, harvested, and plated in 24-well tissue culture plates at 2 × 10^5^ cells/mL. Then, 20 ng/mL of phorbol myristate acetate (PMA) (SERVA, Heidelberg, Germany) was added, and samples were incubated for 24 h at 37 °C in 5% CO_2_ to obtain macrophage-like cells. The cells were then stimulated with *E. coli* lipopolysaccharide B (DIFCO Laboratories) at a final concentration of 50 ng/mL and treated with the test compounds/extracts at three different concentrations (2, 10, and 50 µg/mL). The cells were then incubated for 4 h at 37 °C in 5% CO_2_. The levels of TNF-α in the supernatants were analysed using the human TNF-α DuoSet ELISA (R&D Systems, Minneapolis, NY, USA) according to the manufacturer’s instructions [40]. Pentoxifillin was used as a standard TNF-α inhibitor (IC_50_ = 94.8 ± 2.1 µg/mL).

#### 4.4.4. Lymphocyte Proliferation Assay

The ^3^H-thymidine-incorporated T-cell proliferation assay was performed [41]. Peripheral human blood T-lymphocytes were briefly isolated *via* the Ficoll-Hypaque density gradient method. Cells (at approximately 2 × 10^6^ cells/mL) were incubated with the test compounds at three different concentrations (1, 10, and 100 μg/mL) along with 7.5 μg/mL of phytohemagglutinin (PHA) in 5% RPMI-1640 at 37 °C in a CO_2_ environment for 72 h. All tests were run in triplicate. After continuous incubation for 18 hours following the addition of 25 µL/well of 5 µCi thymidine (^3^H) (Hartmann Analytic, Braunschweig, Germany), the cells were harvested using a cell harvester (Inotech Dottikon, Heerbrugg, Switzerland). The proliferation level was determined *via* a radioactivity count (in CPM) using a Scintillation counter (LS 6500, Beckman Coulter, Fullerton, CA, USA). The IC_50_ values were calculated. Prednisolone was used as standard drug (IC_50_ = <0.62 µg/mL) [41].

## 5. Conclusions

The translation of traditional medicine into evidence-based therapeutic entities warrants continuous investigation and extensive analyses to exploit the feasibility, reliability, and safety of those natural remedies. In this vein, a bioassay-guided isolation of *T. aphylla* L. was carried out followed by evaluation of the immunosuppressive impact of the extracts (*n*-hexane, dichloromethane, ethyl acetate, *n*-butanol, and water) and isolated compounds **1**–**4** from the aerial parts of *T. aphylla* L., validating the traditional uses of this plant in the treatment of inflammatory diseases. Compound **2** caused inhibition of the production of ROS and TNF-α, whereas compound **3** showed inhibitory activity against all tested mediators (NO, ROS, and TNF-α). Better understanding of the potential aspect of *T. aphylla* L. derivatives as anti-inflammatory agents could open the door for the production of advanced anti-inflammatory entities. These results also highlight the importance of this plant as a candidate for the isolation of novel anti-inflammatory products. The *T. aphylla* L. derivatives are rich sources of anti-inflammatory and antioxidant compounds for the future development of nutraceuticals/phytopharmaceuticals.

## Figures and Tables

**Table 1:** Anti-inflammatory parameters as tested by the extracts and compounds isolated from the aerial parts of *T. aphylla* L.

Code	ROS Inhibition (IC_50_ ± SD µg/mL)	Nitric Oxide (NO) Inhibition (IC_50_ ± SD µg/mL)	TNF-α Inhibition (IC_50_ ± SD µg/mL)	T-Cell Proliferation Inhibition (IC_50_ ± SD µg/mL)
**EtOH: H_2_O**	16.7 ± 0.6	29.5 ± 1.7	>100	13.8 ± 2.4
**DCM**	19.2 ± 1.8	14 ± 0.6	3.7 ± 0.3	>100
***n*-BuOH**	19.8 ± 3.5	27.2 ± 0.07	44.9 ± 8.9	8.1 ± 0.3
**EtOAc**	12 ± 0.6	>50	>100	43.3 ± 1.2
**Compound 1**	79.5 ± 6.7	NT	NT	NT
**Compound 2**	3.0 ± 0.2	>50	12.6 ± 0.2	>100
**Compound 3**	2.5 ± 0.8	2.4 ± 0.3	5.5 ± 1.1	22.9 ± 1.5
**Compound 4**	>100	NT	NT	NT
**Ibuprofen**	11.2 ± 1.9	-	-	-
**L-NMMA**	-	24.2 ± 0.8	-	-
**Pentoxifillin**	-	-	94.8 ± 2.1	-
**Prednisolone**	-	-	-	<0.62
